# Low Comparability of Nutrition-Related Mobile Apps against the Polish Reference Method—A Validity Study

**DOI:** 10.3390/nu13082868

**Published:** 2021-08-20

**Authors:** Agnieszka Bzikowska-Jura, Piotr Sobieraj, Filip Raciborski

**Affiliations:** 1 Department of Clinical Dietetics, Faculty of Health Sciences, Medical University of Warsaw, E Ciolka Str. 27, 01-445 Warsaw, Poland; 2 Department of Internal Medicine, Hypertension and Vascular Diseases, Faculty of Medicine, Medical University of Warsaw, Banacha Str. 1a, 02-091 Warsaw, Poland; piotr.sobieraj@wum.edu.pl; 3 Department of Prevention of Environmental Hazards, Allergology and Immunology, Faculty of Health Sciences, Medical University of Warsaw, 02-091 Warsaw, Poland; filip.raciborski@wum.edu.pl

**Keywords:** mobile applications, dietary record, dietary assessment, dietary methodology

## Abstract

Nutrition-related mobile applications (apps) are commonly used to provide information about the user’s dietary intake, however, limited research has been carried out to assess to what extent their results agree with those from the reference method (RM). The main aim of this study was to evaluate the agreement of popular nutrition-related apps with the Polish RM (Dieta 6.0). The dietary data from two days of dietary records previously obtained from adults (60 males and 60 females) were compared with values calculated in five selected apps (FatSecret, YAZIO, Fitatu, MyFitnessPal, and Dine4Fit). The selection of apps was performed between January and February 2021 and based on developed criteria (e.g., availability in the Polish language, access to the food composition database, and the number of downloads). The data was entered by experienced clinical dietitians and checked by one more researcher. The mean age of study participants was 41.7 ± 14.8. We observed that all the apps tended to overestimate the energy intake, however, when considering the macronutrient intake, over- and underestimation were observed. According to our assumed criterion (±5% as perfect agreement, ±10% as sufficient agreement), none of the apps can be recommended as a replacement for the reference method both for scientific as well as clinical use. According to the Bland-Altman analysis, the smallest bias was observed in Dine4Fit in relation to energy, protein, and fat intake (respectively: −23 kcal; −0.7 g, 3 g), however, a wide range between the upper and lower limits of agreement were reported. According to the carbohydrate intake, the lowest bias was observed when FatSecret and Fitatu were used. These results indicate that the leading nutrition-related apps present a critical issue in the assessment of energy and macronutrient intake. Therefore, the implementation of validation studies for quality assessment is crucial to develop apps with satisfying quality.

## 1. Introduction

Noncommunicable diseases (NCDs) (e.g., cardiovascular diseases, diabetes mellitus type 2, obesity) kill 41 million people each year, accounting for about 71% of all deaths worldwide and 90% of deaths in Poland [1,2]. General recommendations for this epidemic require lifestyle changes, which include healthy eating habits, regular physical activity, and the reduction of tobacco smoking and alcohol consumption [3]. Considering unhealthy dietary habits are one of the main risk factors of NCDs, the valid recording of dietary intake may be key for nutritional and clinical interventions. Traditional dietary assessments have relied on interviewer or paper-and-pen-based methods, and in clinical nutrition these methods are usually applied between 3 and 7 days [4].

The recently observed development of mobile technology has led to the growth of novel electronic dietary assessment methods, such as nutrition-related applications (apps), which are commonly used on mobile phones. By using these apps, users can follow their dietary intake with web-based food recalls, track changes in their anthropometric measurements, and receive health tips based on their current lifestyle behavior. The benefits of these new technological tools are the ease of use and logging of food in real-time. In addition, users can set goals to increase their motivation and keep track of their progress [5,6,7]. Undoubtedly, all these advantages are crucial for individuals with NCDs [8,9]. Nonetheless, scientific evidence is still limited in considering the evaluation of data infallibility and the effective use of apps in clinical practice [10]. Therefore, there is a need to evaluate the potential and the limitations of these tools. Regarding the fact that these apps allow the user to self-monitor and track dietary intake, they can also reveal some disadvantages. Incorrect use (e.g., entering food portions on the basis of rough estimation) may cause health problems as well as maintain or trigger eating disorders [11,12,13].

The basis for developing nutrition-related apps are food composition databases (FCDs) which include detailed information about the nutritional composition of foods and are important in different fields, from clinical practice to epidemiological research in the food industry and for educational purposes [14,15]. The energy value and the nutrient content of a given diet is usually calculated with software that is based on available country-specific FCDs. In Poland, Dieta 6.0 software is considered as the reference method (RM) as it is based on the Polish Food Composition Database (National Food and Nutrition Institute, Warsaw, Poland) [16]. It is designed according to well-defined methods, starting from Polish food composition data and foreign sources and ending with scientific papers.

Regarding the source of FCD and the affiliation of app developers and their objectives, two major categories of dietary-related apps may be distinguished. First are the academic apps developed by nutritional experts for research purposes, e.g., My Meal Mate [17] and DietCam [18]. The second group includes consumer-grade apps, typically developed by private institutions specialized in the digital industry, mainly for commercial purposes, e.g., MyFitnessPal, FatSecret [19]. From a scientific point of view, the first ones are more reliable and have more advantages, mostly thanks to scientific input, comparable with the RM. These apps do not aim to be very popular, contrary to consumer-related apps, which aim at increasing the number of users. The use of a large volume of varied FCDs is usually a crucial differentiating factor, as well as a potential source of under- or overestimation in consumer-grade apps [20,21].

Most of the previous validation studies focused on consumer-grade apps have been limited to the countries outside Europe (United States [22,23], Australia [24,25], Japan [26], Brazil [27]) and none have been conducted in Poland. Additionally, considering the constant updating of FCDs, which are the base of nutrition-related mobile apps, there is an urgent and ongoing need for further evaluation. Therefore, regarding the importance of valid dietary intake and technological advancement, the objective of this study was to evaluate the comparative validity of consumer-grade mobile apps in the energy and macronutrient calculations in comparison with the Polish RM. Additionally, we aimed to review the main input and output features of selected mobile apps, which may be useful not only for non-professional users but particularly for academic and health professionals who are interested in nutritional assessment.

## 2. Materials and Methods

### 2.1. Test Nutritional Data and Polish Reference Method

For the test nutritional data inputted into each mobile app, we used information obtained from people who participated in two projects under the National Health Program between the years 2016 and 2020, financed by the Minister of Health. The main aim of both projects was to assess the diet and nutritional status of the Polish population. Both projects were performed on two representative Polish samples (each sample included 2000 people) aged 19–64 and >65 years old. Data was collected from 2017 to 2020. For the purpose of the present analysis, 120 people were randomly selected (out of 4000 for whom data were available). The subjects were divided into 6 strata (sub-groups) determined on the basis of age and gender, and then, 20 people were randomly selected from each layer.

Two-days dietary recalls were obtained from participants involved in the studies mentioned before. Information about all foods and beverages consumed by the study participants for two days before the face-to-face interview was collected. The interviews were performed by trained interviewers. The sizes of declared food portions and meals were verified using the “Album of Photographs of Food Products and Dishes” from the National Food and Nutrition Institute [28]. The data for the Dieta 6.0 were entered by experienced clinical dietitians. All data entered to the Dieta 6.0 were expressed in each food name and gram values.

As it was mentioned before, the Polish RM for assessing the nutritional value of the diets is the Dieta 6.0 software (National Food and Nutrition Institute, Warsaw, Poland), based on the Polish Food Composition Database. The Dieta 6.0 software is a unique computer program designed for planning diets and assessing individual or group nutrition and for current analyses of the consumption of the surveyed population groups in comparison with nutritional recommendations. The main purpose of the Dieta 6.0 program is to enable the calculation of the energy and nutritional value of diets, as well as the amount of food and food consumption, by comparing the calculated nutritional value to the recommendations, and considering the new approach in terms of the adequacy of the assessment of nutrient intake, using the probability and cut-off methods [29,30,31].

### 2.2. Sample Size and Study Outcome

The primary study outcome is the agreement between the tested apps and the reference method according to the energy (kcal). The calculation of sample size was performed similarly to [26], assuming beta = 0.2 and alpha = 0.05, and was performed for the Bland-Altman analysis for energy intake agreement assessment. We assumed an expected mean of differences of 0 kcal, an expected standard deviation of differences of 100 kcal, and a maximum allowed difference between methods of 250 kcal. Using such assumptions, the calculated sample size was 111. Therefore, we selected 120 participants through stratified randomization such that 20 males and 20 females from each of three age categories (19–39, 40–59, ≥60 years) were included.

The secondary outcomes are the agreements between tested apps and the reference methods according to protein, fat, and carbohydrate estimation.

### 2.3. Identification and Selection of Mobile Apps

**Figure 1:**
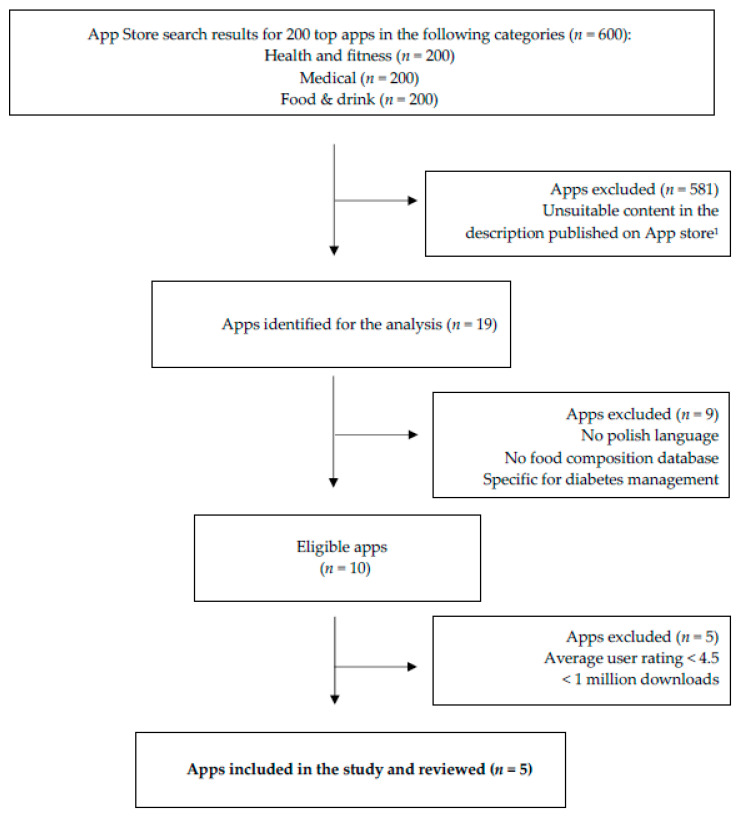
Study flow chart of mobile app selection. ^1^ No possibility to calculate energy and macronutrient intake, no information about the source of food composition database.

The identification and selection of mobile apps were restricted to apps available for free in the App Store for iOS devices available in Poland. Our research strategy was based on the fact that free apps are widely used with many downloads and that the App Store and Google Play Store offerings overlap. We identified 600 ranked apps in the Top Download sections of the three categories: ‘Health and Fitness’ (*n* = 200), ‘Food and Drink’ (*n* = 200), and ‘Medical’ (*n* = 200) between January and February 2021. After an initial screening based on the descriptions provided by the store, we selected 19 apps (581 were excluded). The inclusion criteria were as follows: the tracking of energy and macronutrient intake [26], available in the Polish language, free version available [32], defined FCD). For the remaining 10 mobile apps, we developed the following exclusion criteria <4.5 star-rating and <1 million downloads [13] and finally identified five apps which were analyzed (Figure 1).

### 2.4. The Assessment of Nutritional and Technological Features of Mobile Apps

Once the mobile apps were installed (all on the same device—iPhone 7), their input and output features were reviewed from two perspectives: nutritional and technological. Features that required data from the user (e.g., gender, anthropometric measurements) were considered as input, whereas the results shown to the user were termed output. Considering the nutritional issue, the following issues were investigated: dietary intake, users’ data, and physical activity, along with others (e.g., personal reminders and notes). The technological perspective analyzed what technologies were being used in order to compare with emerging technologies in the field of nutrition evaluation and intervention. The functionalities were analyzed in two main groups: input and output features. The selected apps were: FatSecret, YAZIO, Fitatu, MyFitnessPal, and Dine4Fit. All of the apps were in the ‘Health and Fitness’ category.

### 2.5. Input of Nutritional Data into Mobile Apps

The dietary data were entered by a clinical dietitian between March and April 2021. The food products and mixed meals eaten by each of the participants, except for drinking natural water and dietary supplements, were entered into each of the five apps and then verified by one more researcher. For specific brand food products, the apps’ databases were searched by the name of the brand, and the directly matched item was selected. If we did not recognize any match, a similar generic food or dish was selected. Generic mixed dishes were searched by the dish name (e.g., pork stew) or main food ingredients (e.g., pork shoulder) and an item that best represented the food preparation method or food ingredients was chosen. For each app, the food data were entered selectively, e.g., toast with ham was entered as bread, butter, and ham.

The data implemented to Dieta 6.0 software were expressed in grams, so there were no difficulties in the adjustment of the portion sizes of selected food in the mobile apps. The same gram values as it was in the RM, were entered into each app. Estimated dietary intakes (energy and macronutrients) of each participant calculated by the apps, were manually entered into Excel files. MyFitnessPal has a function for exporting data as CSV (comma-separated values) files, however, due to further statistical analysis, we decided not to use this convenience.

### 2.6. Statistical Analysis

**Figure 2:**
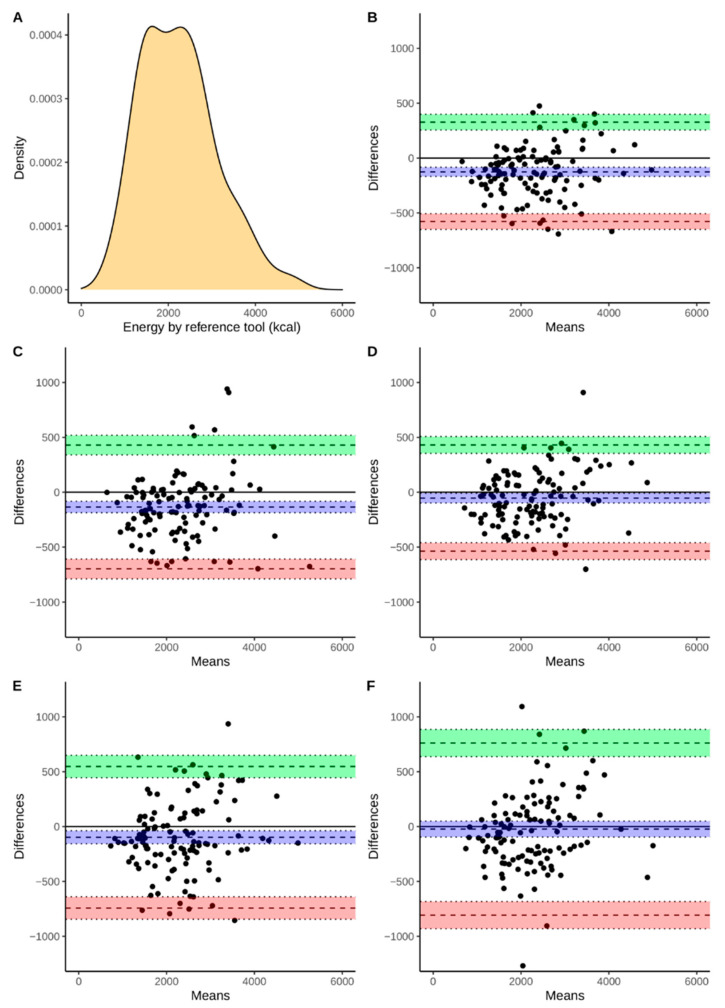
Density of energy intake measured using the RM (Panel **A**), Bland-Altman plot presenting the agreement between FatSecret (Panel **B**), Yazio (Panel **C**), Fitatu (Panel **D**), MyFitnessPal (Panel **E**), Dine4Fit (Panel **F**). Bias, upper and lower limit of agreement are marked using dashed lines; blue, green, and red colors were used to highlight 95% confidence intervals, respectively.

**Figure 3:**
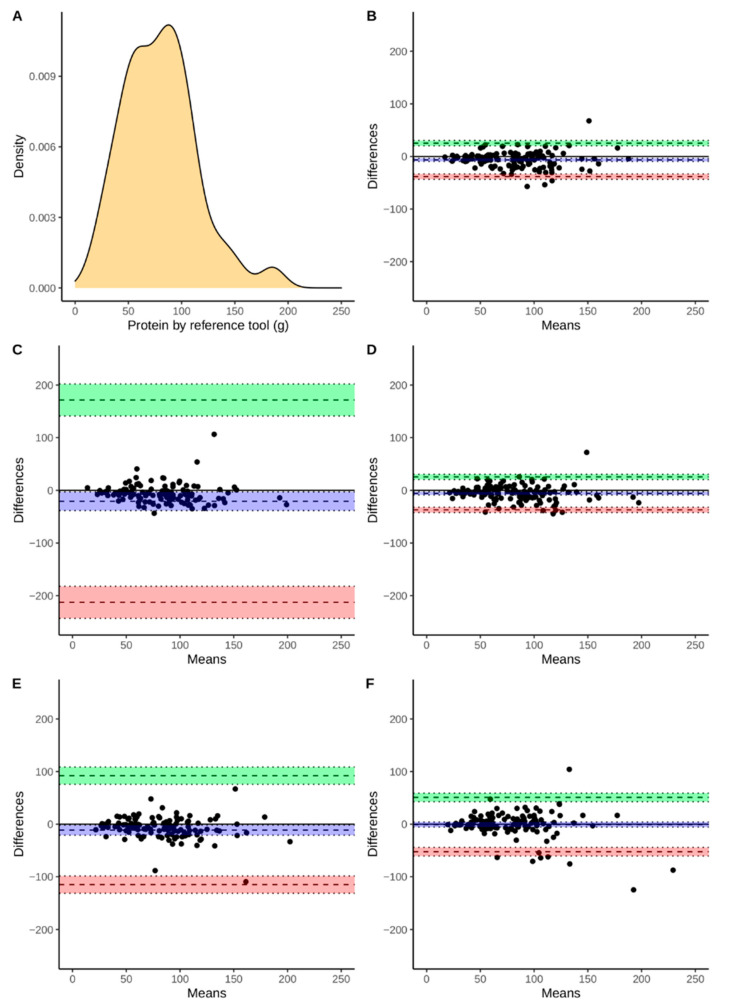
Density of protein intake measured using reference tool (Panel **A**), Bland-Altman plot presenting the agreement between FatSecret (Panel **B**), Yazio (Panel **C**), Fitatu (Panel **D**), MyFitnessPal (Panel **E**), Dine4Fit (Panel **F**). Bias, upper and lower limit of agreement are marked using dashed lines; blue, green, and red colors were used to highlight 95% confidence intervals, respectively.

**Figure 4:**
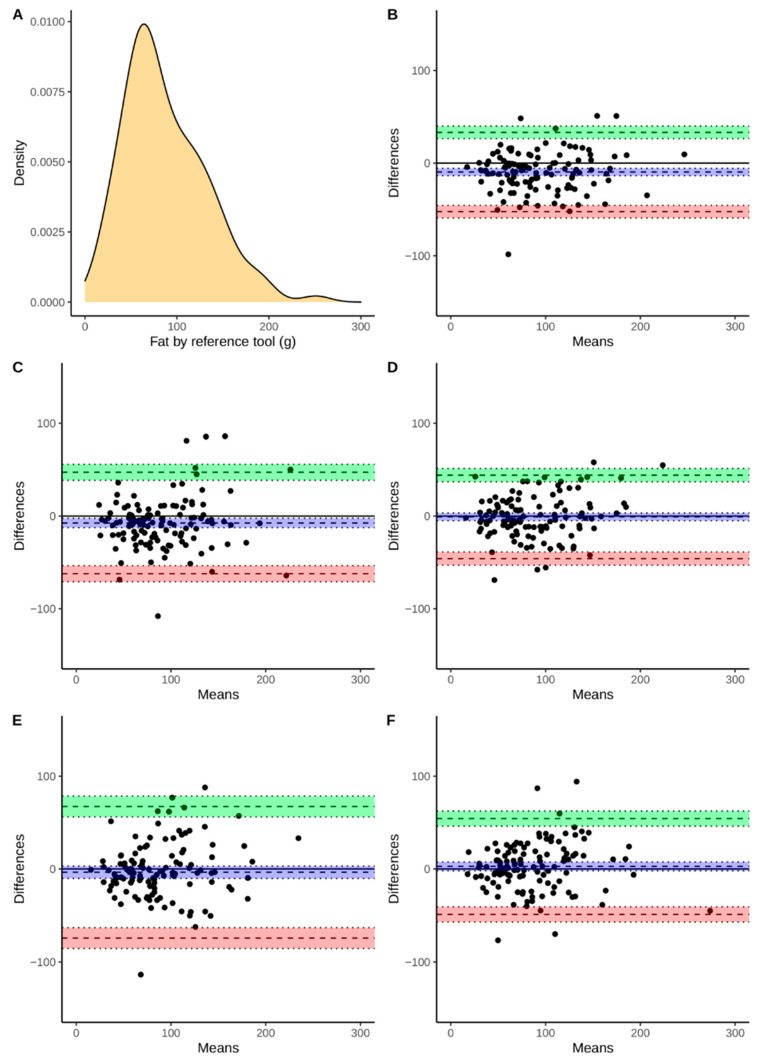
Density of fat intake measured using reference tool (Panel **A**), Bland-Altman plot presenting the agreement between FatSecret (Panel **B**), Yazio (Panel **C**), Fitatu (Panel **D**), MyFitnessPal (Panel **E**), Dine4Fit (Panel **F**). Bias, upper and lower limit of agreement are marked using dashed lines; blue, green, and red colors were used to highlight 95% confidence intervals, respectively.

**Figure 5:**
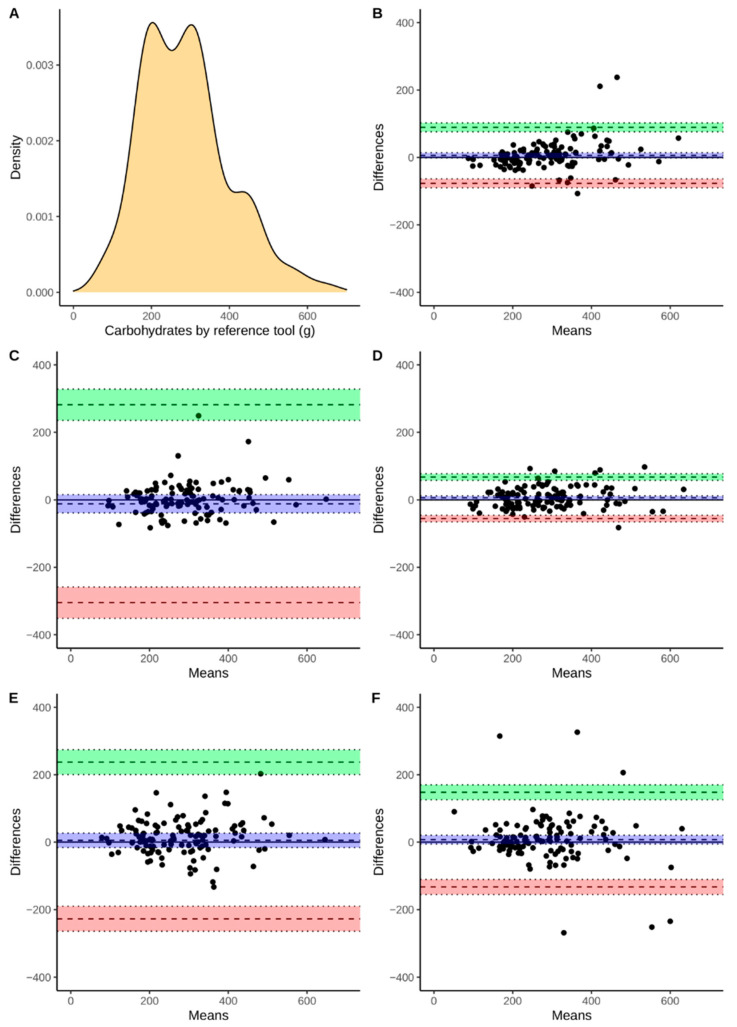
Density of carbohydrate intake measured using reference tool (Panel **A**), Bland-Altman plot presenting the agreement between FatSecret (Panel **B**), Yazio (Panel **C**), Fitatu (Panel **D**), MyFitnessPal (Panel **E**), Dine4Fit (Panel **F**). Bias, upper and lower limit of agreement are marked using dashed lines; blue, green, and red colors were used to highlight 95% confidence intervals, respectively.

Continuous variables were presented using means and standard deviations as well as medians with an interquartile range. Discrete variables are presented as a number followed by percentages. Additionally, Figure 2, Figure 3, Figure 4 and Figure 5 present the density plots of energy, protein, fat, and carbohydrates according to the reference method values. Density plots are used to present the probability of the variable falling within a particular range of values. The normality of the distributions of the analyzed variables was assessed using the Shapiro-Wilk test, when analyzed variables did not have a normal distribution, nonparametric tests were used. For this reason, comparisons were made using the paired Wilcoxon test. Bonferroni correction was used for multiple comparisons. The Spearman correlation coefficients were calculated. The agreement assessment was based on Bland-Altman statistics and plots [33]. Biases (computed as means of differences between the reference method and derived from apps) and lower (LLoA) and upper (ULoA) limits of agreement with 95% confidence intervals were calculated. We have arbitrarily determined a perfect and sufficient agreement. A perfect agreement was classified as when the results obtained by the evaluated app were within ±5% of the value from the reference tool. A threshold of ±10% was chosen to assess the agreement as sufficient. All computations were performed using R, environment for statistical computations (version 4.0.5, The R Foundation for Statistical Computing, Vienna, Austria).

## 3. Results

### 3.1. General Characteristics of Study Participants and Selected Nutrition-Related Apps

The data of 120 subjects, including 60 females and 60 males, were analyzed. The median and interquartile range for age were 41 (28.8–54) years and for BMI 24.7 (22.4–27.7) kg/m^2^. One third (*n* = 40) were overweight (BMI ≥ 25.0 kg/m^2^), 16 subjects (13.3%) were obese (BMI ≥ 30.0 kg/m^2^), while 1 (0.8%) had BMI <18.5 kg/m^2^ (Table 1).

In Table 2 and Table 3 the input and output features of all apps are presented. Both types of features presented for all evaluated apps indicated that most of the elements were common in all of them. Considering the dietary input features, YAZIO and FatSecret have all presented functionalities, however, in FatSecret three of them (water consumption, food images, and adding own dishes) were available only in the premium (paid) version. The most frequent phenotype inputs were gender, age (calculated from birthdate), weight, and height, whereas circumferences (e.g., hips, waist) were found only in two apps (MyFitnessPal and Fitatu) and entered optionally after the initial registration. One app (Dine4Fit) offered the possibility of daily notes and none of the apps required information about health conditions.

Output features concerning nutritional assessment were similar in all apps in terms of energy and macronutrient intake. All apps provided information on selected micronutrient intakes and three apps (YAZIO, Fitatu, MyFitnessPal) calculated vitamin intake. The user’s weight progress shown in graphs or charts was found in all apps, however, BMI calculation was available only in one app (Dine4Fit).

### 3.2. Comparison of Energy and Macronutrient Intake between Apps and Polish RM

The macronutrient and energy contents of the diets were assessed using the RM and five evaluated applications in all subjects. The median and 2nd and 3rd quartiles were calculated and presented in Table 4. Half of the comparisons showed that significant differences can be found between values calculated by evaluated apps and the RM. The exception was Dine4Fit, for which no significant differences were found (*p* > 0.05).

Strong (r > 0.6) and very strong (r > 0.8) correlations were found between values estimated by evaluated apps and the RM (Table 5). All correlations were significant (*p*-value < 0.001).

### 3.3. The Agreement between Selected Mobile Apps and RM

The agreement between the assessment of energy and macronutrient intake evaluated by apps and the RM was assessed using Bland-Altman plots (Figure 2, Figure 3, Figure 4 and Figure 5). According to the energy intake (Figure 2B–E), the biases were below 0 kcal in all of the applications. However, the range between the lower and upper limits of the agreement may be considered as large. The smallest range was observed for FatSecret and the highest for the Dine4Fit app. The smallest bias according to the protein intake (Figure 3B–E) was observed when Dine4Fit was used. Yazio had the highest range between the lower and upper limits of the agreement. The smallest range between the lower and upper limits of the agreement was observed when protein intake was assessed using FatSecret and Fitatu. According to the fat intake (Figure 4B–E), FatSecret had the smallest range between the lower and upper limits of agreement. However, FatSecret had the highest bias. The lowest bias was found when Fitatu was used to assess the fat intake. The highest bias and the highest range between the lower and upper limits of agreement was observed when Yazio was used to assess the carbohydrates intake (Figure 5B–E). MyFitnessPal had the smallest bias, while Fitatu had the smallest range between the lower and upper limits of the agreement. The exact values of biases, lower, and upper limits of agreement followed by 95% confidence intervals are presented in Table 6.

The agreement between evaluated apps and the RM is also assessed according to the predefined agreement criteria (±5% as perfect agreement, ±10% as sufficient agreement). Regarding energy intake, the agreement was rated as perfect in 43 (35.8%), 45 (37.5%), 34 (40.8%), 24 (20%), and 28 (23.3%) subjects when FatSecret, YAZIO, Fitatu, MyFitnessPal, Dine4Fit were used, respectively. The perfect agreement in estimating protein intake was less frequent: 35 (29.2%), 21 (17.5%), 26 (21.7%), 22 (18.3%), and 22 (18.3%), respectively. When agreement in assessment of fat intake was rated, the perfect agreement was observed in 22 (18.3%), 20 (16.7%), 15 (12.5%), 19 (15.8%), and 15 (12.5%) subjects. The perfect agreement was observed more often when carbohydrates intake was assessed, respectively, in 49 (40.8%), 38 (31.7%), 37 (30.8%), 27 (22.5%), and 30 (25%) cases. Data concerning “sufficient agreement” was presented in Table 7.

## 4. Discussion

This study indicated that, overall, in comparison to the Polish RM, the most popular nutrition-related apps tended to overestimate energy intake. However, when considering macronutrient intake, over- and underestimation was observed. In most cases, dietary intake calculated with mobile apps was affected by an error greater than ±5%. Regarding energy intake, the agreement was rated as perfect only in 20 to 40.8% of subjects. When less restrictive criteria (agreement at the level of ±10%) were applied, the results still remained unsatisfactory (48.3 to 60.8% of all subjects).

For the estimation of energy intake, Dine4Fit demonstrated the best agreement with the RM, evidenced by small bias (−23 kcal; −93 to 50 kcal) and a strong correlation coefficient (r = 0.86), however, concerning predefined perfect agreement criteria (± 5%), the output was one of the worst (*n* = 28, 23.3%) as well as a wide range between lower and upper limits of agreement being present. Similarly, no satisfying agreement was found between macronutrient intake and the RM. Regarding protein intake, the lowest bias was observed in Dine4Fit (−0.7 g; −5 to 5 g) and the highest was in YAZIO (−21 g; −38 to 3 g), whereas for fat intake it was in Fitatu (−0.9 g; −5 to 4 g) and in FatSecret (−10 g; −14 to −6 g), respectively. Concerning the calculations of carbohydrate intake, these values were in the best agreement with the RM, and in almost 41% of cases (FatSecret), we reported a perfect agreement.

To the best of our knowledge, this is the first comparative validity study in Poland, so we could compare our results only with reports from other countries. Similar research was performed in the United Kingdom, where Fallaize, et al. [32] compared popular nutrition-related apps with the British RM (DietPlan6). The main observation of this study was that the estimation of energy and saturated fat intake between the RM and diet apps was not different. Simultaneously, the authors indicated that estimates of protein were significantly lower using FatSecret than using the RM. This finding is consistent with our results, using the same app, bias for protein was −7 (−9 to −4). In turn, Griffiths, et al. [22] observed that selected mobile apps (MyFitnessPal was the only one in common with our apps) tended to underestimate the intake of most nutrients and that comparative validity of energy calculations to Nutrition Data System for Research (an American Windows-based dietary analysis software) may be better than the estimate for macronutrients. In another study performed in Japan [26], one of the apps that was analyzed was MyFitnessPal, involved also in our analysis. Japanese researchers indicated that in comparison to other selected apps (*n* = 4), MyFitnessPal had greater mean differences and wider limits of agreement. Additionally, the authors also chose four food items that existed in all apps and had a high consumption frequency in the 4-day dietary recalls and compared their nutritional value calculated using apps and the RM. Interestingly, for white rice, MyFitnessPal overestimated the energy and macronutrient content, up to a maximum of 200% difference (for total fat). These findings suggest that all dishes containing white rice would be overestimated (with the exception of dishes for which the total energy value has been entered manually). Contrary to our results, Tosi, et al. [13] indicated that all selected nutrition-related apps (*n* = 5), three of them were used in our study, (YAZIO, FatSecret, and MyFitnessPal) tended to underestimate energy intake compared with the RM (3-day food diary), whereas the highest underestimation was observed in YAZIO (−329 kcal). Moreover, long-term use of this app could lead to the chronic inaccuracy of energy intake (e.g., −768 kcal over 7 days). Considering the energy restrictions recommended in overweight or obese patients (deficit of 300–500 kcal/day) [34], values calculated with YAZIO should be treated with caution, as not to lead to an excessive deficit of energy. Interestingly, Chen, et al. [35] found that the energy values derived from MyFitnessPal were significantly underestimated in comparison to two- and four-day dietary recalls and amounted to −435 kcal (SD 705.5 kcal, *p* = 0.0002), which corresponds to the energy deficit indicated in weight reduction. On the other hand, Simpson and Mazzeo [11] explored associations between the use of energy-tracking apps and eating behaviors among students. Individuals who declared the use of these apps presented higher levels of eating concern and dietary restraint. For this reason, the authors suggested that people with obesity or eating disorders connected with an excessive amount of food consumption (e.g., binge eating) should be encouraged to use these technological facilities in accordance with professional recommendations.

The cause of over- or underestimation of dietary intake revealed in our study may be due to various reasons. Despite the fact that the experienced clinical dietitian entered the data into mobile apps, a huge number of food options can be oppressive and can compromise the appropriate selection of correct food products, as was reported by Chen, et al. [35]. Moreover, although the presence of a crowd-sourced database may be beneficial for increasing the number of food items, given that users can upload them by entering only the food name, some energy and nutrient values may be incorrect or missing [19,32,36]. Additionally, as we have mentioned before, the use of different FCDs could be also prone to error for energy and nutrients intake estimation [37]. In fact, another reason for under- or overestimation may be due to the possibility that users of apps may enter their own composition of nutritional values. Although the base for the mobile apps analyzed in the present study were different FCDs, all apps also used crowd-sourced databases. The expanding and the modifiability of FCDs offer the benefit of the customization of foods consumed, but on the other hand present drawbacks in the validity of nutrients estimation. When app users are allowed to enter the nutritional values on the labels, they may insert typos leading to losses of the original quality of FCD, as in the case of buckwheat (100 g = 84 kcal reported by Dine4Fit compared with 336 kcal reported by RM). These errors may significantly affect validity in the calculation of energy and nutrient intake, introducing additional sources of variation, hence, a precise verification of data uploaded by a dietitian/nutritionist should be demanded. Additionally, regarding the real-life use of mobile apps, the expected causes of inappropriate estimation are different and mainly involved the wrong selection of food items and incorrect determination of portion size.

Apart from the calculation of dietary intake, in the present research, we present selected input and output features of all selected apps. This evaluation could be a source of information for professionals and users about specific features and the strengths/weaknesses of the most popular apps. Considering this issue, Chen, et al. [38] performed a study that aimed to identify dietitians’ user preferences concerning the tools, design features, and resources of mobile apps that would support their patients in following dietary recommendations. They found that for dietitians, the most important features of the apps were credibility and usability. In another study, Chen, et al. [35] reported that among the Australian, New Zealand, and British Dietetic Associations the most often recommended nutrition-related apps were MyFitnessPal (involved in our study) and the Monash University Low FODMAP Diet.

In our study, all analyzed apps enabled weight control, whereas none of them required information about the user’s health status. This observation suggests that in all apps there is a general focus on weight loss and calorie counting. Previous studies revealed that apps dedicated to weight reduction or management are of suboptimal [24] or low [39] quality. Chen, et al. [24] aimed to assess the quality of the most popular smartphone apps for weight loss (*n* = 28) and found that only one app (4%) met all accountability criteria. Despite the fact that most of the included apps achieved good results for scientific coverage and accuracy, the scientific scope of weight management information has been limited. In turn, Nikolau, et al. [39] analyzed 28,905 apps and reported that only 0.05% of them (*n* = 1445) were developed with professional sources. Interestingly, Pagoto, et al. [40] observed that apps focusing on weight reduction typically included only a few behavioral strategies found in interventions based on evidence. Behavioral strategies that help increase motivation, reduce stress, and assist with problem-solving were missing across apps. The authors concluded that the inclusion of additional strategies could make apps more helpful to users who have motivational challenges. On the other hand, systemic reviews and metanalysis [9,41,42] performed in past years revealed that dietary interventions including self-monitoring based on nutrition-related apps may be a useful tool for weight management or reduction. Flores Mateo, et al. [9] analyzed 12 articles and observed that the use of a mobile app was associated with significant changes in body weight (kg) and body mass index (kg/m^2^), −1.04 kg and −0.43 kg/m^2^, respectively. While these were positive results, they should be interpreted with caution, as there were different control interventions (e.g., brochures/booklets, nutritional education, intensive counseling), varied duration of the studies, and small sample size [9,42]. However, it is important to note that nutrition assessment should not be only related to weight control, but also to different clinical situations which require the implementation of specific dietary recommendations. In those cases, the utility of evaluated mobile apps may be limited. This mainly applies to diseases in which dietary intake of selected vitamins and minerals is more important than the energy value of the diet (e.g., anemia, osteoporosis, hyperhomocysteinemia). Regarding apps selected in the present study, three out of five calculated vitamin intake, and for MyFitnessPal it was only for vitamin A and C. FCDs usually contain few products for special nutritional purposes, therefore the usability of mobile apps is also limited to people requiring clinical and elimination diets.

Some methodological limitations of this study should be mentioned. Firstly, the dietary data was entered into each app by the clinical dietitian, whereas the average person with significantly lower nutritional knowledge may find it more difficult to match appropriate food products or to define which food portion or how many grams should be entered. Secondly, we did not perform the analysis of the current use of apps in a real-life setting and before selecting participants’, we did not conduct sensitivity analysis, therefore there is a risk of selecting people who were over- or under-reporters. Furthermore, we provided results only for energy and macronutrient calculations. Moreover, the agreement criteria (5% and 10%) were proposed by us and are not supported by the evidence yet. Finally, we analyzed the validity of apps available for free in the App Store, for this reason, other relevant paid apps or those distributed in other stores such as Google Play may have been left out.

One of the main strengths of our study was a large quantity of nutritional data from 240 dietary recalls reported by 120 study participants, that were entered into five mobile apps. Additionally, considering the ‘Health and Fitness’ category, three of the four evaluated apps were in the top 100 grossing apps in Poland, which may suggest that our results may be significant for many users of nutrition-related apps. Moreover, our study presents not only the thorough examination of energy and macronutrient calculations, but also other features (input and output) of the apps. This evaluation could be a source of information for professionals and users about specific features and the strengths/weaknesses of the most popular apps.

## 5. Conclusions

Overall, our findings suggest that dietitians and other health professionals should be careful in recommending the use of popular nutrition-related apps and these apps should not be used in research settings. As presented in the current study, the leading diet-tracking apps present critical issues in calculating energy and macronutrient intake, therefore, app developers should consider implementing testing plans to quickly identify issues with their app that require improvement in the accuracy of dietary intake estimation. Another important issue to consider is the ongoing verification of nutritional data entered by the users. Then, after checking the entered values by the professionals, a given product or meal should be included in the database and be visible to other users. It would also be a good idea to provide information about the source for the nutritional value, if it was for example a USDA SR or food product label. Undoubtedly, further research on the validity and the use of apps to monitor food consumption is also desirable.

## Figures and Tables

**Table 1:** Characteristic of study participants.

	All Subjects, *n* = 120	Men, *n* = 60	Women, *n* = 60
Age (years)	41 (28.8–54)	40 (29–53)	44 (28–54)
BMI^2^ kg/m^2^	24.7 (22.4–27.7)	25 (23–28)	24 (22–28)
BMI < 18.5 kg/m^2^	1 (0.8%)	0 (0%)	1 (1.7%)
BMI ≥ 18.5 and <20 kg/m^2^	63 (52.5%)	29 (48.3%)	34 (56.7%)
BMI ≥ 20 and <25 kg/m^2^	40 (33.3%)	24 (40%)	16 (26.7%)
BMI ≥ 30 kg/m^2^	16 (13.3%)	7 (11.7%)	9 (15%)

Age and BMI values are presented as median and interquartile range BMI—body mass index.

**Table 2:** Input features of nutrition-related apps.

Feature/App	FatSecret	YAZIO	Fitatu	MyFitnessPal	Dine4Fit
Dietary features					
The source of FCD ^1^	USDA SR ^2^ Australian FCD ^1^ Crowd-sourced	USDA SR ^2^ BLS ^3^ Crowd-sourced	USDA SR ^2^ Polish FCD ^1^ Crowd-sourced	USDA SR ^2^ Crowd-sourced	Czech FCD ^1^
Number of food items	34,000	No data	>1,600,000	>300,000,000	934
Barcode scanner	✓	✓	✓	✓	✓
Branded food products	✓	✓	✓	✓	✓
Serving size	✓	✓	✓	✓	✓
Favorite foods	✓	✓	✓	✓	✓
Uploading own data ^4^	✓	✓	✓	✓	✓
Water consumption	✓ ^5^	✓	-	✓	✓
Food images	✓ ^5^	✓	✓	-	✓
Adding own dishes	✓ ^5^	✓	✓	✓	✓
Setting dietary goals	✓	✓	✓	✓^5^	✓
Users’ data					
Gender	✓	✓	✓	✓	✓
Weight	✓	✓	✓	✓	✓
Height	✓	✓	✓	✓	✓
Circumferences	-	-	✓	✓	-
Birth date	✓	✓	✓	✓	✓
Physical activity					
Type of physical activity	✓	✓	✓	✓	✓
Setting exercise goal	-	✓	✓	✓	-
Average activity level	✓	✓	-	✓	-
Other features					
Daily notes	-	✓	-	-	✓
Health status	-	-	-	-	-
Personal reminders	-	-	-	✓	✓
Community forums	✓	✓	-	✓	-
Keeping track of progress	-	✓	✓	✓	-

^1^ FCD, food composition database. ^2^ USDA SR, US Department of Agriculture National Nutrient Database for Standard Reference. ^3^ BLS, Bundeslebensmittelschlüssel, The German Nutrient Database ^4^ Users may freely upload the nutritional value of their own food items which are not available in the app. ^5^ Available in premium (paid) version.

**Table 3:** Output features of nutrition-related apps.

Feature/App	FatSecret	YAZIO	Fitatu	MyFitnessPal	Dine4Fit
Automated nutritional assessment					
Total energy intake	✓	✓	✓	✓	✓
Energy intake by meal	✓	✓	✓	✓	✓
Macronutrient intake	✓	✓	✓	✓	✓
Micronutrient intake	✓ ^1^	✓	✓	✓ ^2^	✓ ^3^
Vitamins intake	-	✓	✓	✓ ^4^	-
Recommended water consumption	-	✓	✓ ^5^	-	✓
Diet plan	✓ ^5^	✓	✓ ^5^	-	-
Shopping list	-	-	✓ ^5^	-	-
CVS file with nutrition data	-	-	✓ ^5^	✓^5^	-
Other features					
Weight changes	✓	✓	✓	✓	✓
BMI ^1^ calculation	-	-	-	-	✓
Energy expenditure	✓	✓	✓ ^5^	✓	✓
Private social media	✓	✓	-	✓	-
Sharing with professionals	✓	-	-	-	-

^1^ Data only for sodium and potassium. ^2^ Data only for sodium, potassium, calcium, and iron. ^3^ Data only for calcium and sodium. ^4^ Data only for vitamin A and C. ^5^ Available in premium (paid) version.

**Table 4:** Descriptive characteristics of energy and macronutrient intake as estimated by the RM and selected mobile apps.

	Variables	Median (IQR) ^1^	*p*-Value ^3^
RM ^2^	Energy (kcal)	2193 (1504–2767)	-
	Protein (g)	80 (55–100)	-
	Fat (g)	76 (55–117)	-
	Carbohydrates (g)	281 (198–339)	-
FatSecret	Energy (kcal)	2292 (1695–2865)	<0.001
	Protein (g)	89 (57–108)	<0.001
	Fat (g)	89 (66–130)	<0.001
	Carbohydrates (g)	271 (208–335)	0.972
YAZIO	Energy (kcal)	2320 (1703–2825)	<0.001
	Protein (g)	88 (56–110)	<0.001
	Fat (g)	92 (66–117)	<0.001
	Carbohydrates (g)	278 (208–356)	1.0
Fitatu	Energy (kcal)	2310 (1638–2798)	0.059
	Protein (g)	84 (57–108)	<0.001
	Fat (g)	81 (58–117)	1.0
	Carbohydrates (g)	264 (202–338)	0.587
MyFitnessPal	Energy (kcal)	2328 (1640–2812)	0.003
	Protein (g)	86 (56–112)	<0.001
	Fat (g)	88 (59–107)	0.753
	Carbohydrates (g)	263 (197–340)	0.018
Dine4Fit	Energy (kcal)	2221 (1658–2744)	0.549
	Protein (g)	81 (52–98)	1.0
	Fat (g)	80 (55–108)	1.0
	Carbohydrates (g)	260 (202–335)	0.578

^1^ IQR, Interquartile range. ^2^ RM, reference method. ^3^ The difference from values derived from RM was tested by Wilcoxon signed-rank test.

**Table 5:** Spearman correlation coefficients ^1^ for the association between energy and macronutrient intake as estimated by the RM and selected mobile apps.

	FatSecret	YAZIO	Fitatu	MyFitnessPal	Dine4Fit
Variables					
Energy	0.96	0.95	0.96	0.92	0.86
Protein	0.90	0.83	0.90	0.86	0.82
Fat	0.86	0.81	0.86	0.74	0.80
Carbohydrates	0.95	0.85	0.95	0.86	0.85

^1^ All correlations coefficient were significant (*p* < 0.05).

**Table 6:** Results of Bland-Altman analysis presenting the agreement between evaluated apps and RM. Bias, upper limits of agreement (ULoA), and lower limits of agreement (LLoA) are followed by 95% confidence intervals, respectively.

	FatSecret	YAZIO	Fitatu	MyFitnessPal	Dine4Fit	
Energy (kcal)	−126	−135	−53	−97	−23	
	(−167 to −84)	(−187 to −83)	(−98 to −9)	(−157 to −38)	(−95 to 50)	
	327	429	431	548	762	
	(256 to 399)	(340 to 519)	(354 to 507)	(446 to 649)	(638 to 886)	
	−579	−699	−538	−742	−807	
	(−650 to −507)	(−788 to −610)	(−614 to −461)	(−844 to −640)	(−931 to −683)	
Protein (g)	−7	−21	−6	−11		−0.7
	(−9 to −4)	(−38 to −3)	(−9 to −3)	(−21 to −2)		(−5 to 4)
	25	172	26	92		51
	(20 to 30)	(141 to 202)	(21 to 31)	(76 to 109)		(43 to 59)
	−38	−213	−37	−115		−52
	(−43 to −33)	(−243 to −182)	(−42 to −32)	(−131 to −99)		(−60 to −44)
Fat (g)	−10	−7	−0.9	−4		3
	(−14 to −6)	(−13 to −2)	(−5 to 3)	(−10 to 3)		(−2 to 7)
	33	47	44	67		54
	(26 to 40)	(39 to 56)	(37 to 51)	(56 to 78)		(46 to 62)
	−52	−62	−46	−74		−49
	(−59 to −46)	(−71 to −54)	(−53 to −39)	(−85 to −63)		(−57 to −41)
Carbohydrates (g)	6	−12	6	5	7	
	(−1 to 14)	(−39 to 15)	(0.4 to 12)	(−16 to 27)	(−6 to 20)	
		282	68	237	148	
	89	(235 to 328)	(58 to 77)	(201 to 274)	(126 to 170)	
	(76 to 102)					
		−305	−56	−227	−133	
	−77	(−351 to −258)	(−65 to −46)	(−264 to −190)	(−155 to −111)	
	(−90 to −64)					

**Table 7:** The agreement between evaluated apps and RM using “sufficient agreement” criterion (±10%) (*n* = 120). Numbers followed by percentages present how often energy and macronutrient values were within the assumed range (±10%) compared to the RM.

	FatSecret	YAZIO	Fitatu	MyFitnessPal	Dine4Fit
Energy	73 (60.8%)	67 (55.8%)	77 (64.2%)	58 (48.3%)	60 (50%)
Protein	58 (48.3%)	41 (34.2%)	52 (43.3%)	40 (33.3%)	49 (40.8%)
Fat	40 (33.3%)	39 (32.5%)	36 (30%)	35 (29.2%)	31 (25.8%)
Carbohydrates	81 (67.5%)	61 (50.8%)	72 (60%)	53 (44.2%)	61 (50.8%)

## Data Availability

The data presented in this study are available on request from the corresponding author. The data are not publicly available due to privacy restrictions.
